# GOLPH2 expression in renal cell cancer

**DOI:** 10.1186/1471-2490-8-15

**Published:** 2008-11-11

**Authors:** Florian Rudolf Fritzsche, Mark-Oliver Riener, Manfred Dietel, Holger Moch, Klaus Jung, Glen Kristiansen

**Affiliations:** 1 Institute of Surgical Pathology, UniversitätsSpital Zürich, Zurich, Switzerland; 2 Berlin Institute for Urologic Research, Berlin, Germany; 3 Institute of Pathology, Charité – Universitätsmedizin Berlin, Berlin, Germany

## Abstract

**Background:**

Renal cell carcinomas (RCC) are among the most common and most lethal genitourinary malignancies. GOLPH2 (golgi phosphoprotein 2, GOLM1) has recently been proposed as a biomarker for hepatocellular and prostate cancer. In this study we analysed the expression patterns and the prognostic and diagnostic value of GOLPH2 in RCC.

**Methods:**

GOLPH2 protein expression was analysed by immunohistochemistry in 104 clinically well characterized RCC cases in comparison with matched normal kidney tissue and in association with clinico-pathological parameters. Statistical analyses including Kaplan Meier analyses were performed with SPSS version 15.0.

**Results:**

GOLPH2 was highly expressed in normal renal tubules and in almost half of RCC with a statistically significant predominance in the papillary and chromophobe histological subtypes. No other associations with clinico-pathological parameters were detectable. The Kaplan-Meier curves showed a weak trend for unfavourable prognosis of tumours with high GOLPH2 expression, but failed significance.

**Conclusion:**

GOLPH2 protein is expressed in normal renal tissue (especially in distal tubular epithelia) and is down-regulated in the majority of clear cell RCC. In papillary and chromophobe RCC GOLPH2 expression is consistently present. In contrast to its diagnostic value in hepatocellular and prostatic carcinomas, a prognostic or diagnostic value of GOLPH2 in RCC appears to be unlikely.

## Background

Renal cell cancer (RCC) is one of the most common genitourinary malignancies and causes of cancer associated death in the United States of America in 2008 [1]. Although conventional tumour parameters like nodal status, existence of systemic metastasis or pT-status are important prognostic factors, new molecular markers are warranted to provide more information on the tumour biology, allowing for a better prognostic and possibly predictive stratification of patients.

GOLPH2 is a golgi phosphoprotein (also known as GP73) of yet unknown function. The 73 kDa Golgi apparatus associated protein is coded by the *GOLM1 *gene on chromosome 9q21.33, first described by Kladney et al. in liver tissue of a patient with giant-cell hepatitis [2]. Structurally, GOLPH2 protein has of a short cytoplasmic N-terminal domain, a membrane-spanning region, some coiled-coil domains and a longer luminal C-terminal domain. The structure includes several areas of possible glycosylation. Due to its localisation at the Golgi appartus the proposed functions include protein modification, cell signalling, intracellular transporting function or mere local structural tasks.

Until now only few studies on GOLPH2 exist. In liver diseases GOLPH2 has been described as a potential serum marker of hepatocellular carcinoma [2-6]. Recently GOLPH2 mRNA has been described in a marker combination to detect prostate cancer from urine samples and soon afterwards two independent studies described GOLPH2 as a prostate cancer tissue marker [7-9].

In this study, we carefully analysed the GOLPH2 protein expression in a well characterized renal cell cancer cohort with matched normal tissue. Central aim was to evaluate the potential diagnostic and prognostic value of GOLPH2. We found GOLPH2 differentially expressed between normal and malignant renal tissue and between the different RCC subtypes, but a prognostic value could not be detected.

## Methods

### Patients

One-hundred-four patients (81 men, 23 women) diagnosed for renal cancer at the Institute of Pathology, Charité – Universitätsmedizin Berlin between 2003 and 2005 were enclosed in this study. The study has been approved by the Charité University Ethics Committee under the title "Retrospective Untersuchungen von Gewebeproben mittels immunhistochemischer Färbung und molekularbiologischer Methoden" ("Retrospective analysis of tissue samples by immunohistochemistry and molecular biological methods" (EA1/06/2004) on 20^th ^September 2004.

Patient age ranged between 28 and 92 years with a median of 62. Histological diagnosis was established according to the guidelines of the World Health Organization. Cases were selected according to tissue availability and were not stratified for any known preoperative or pathological prognostic factor. 83 (79.8%) patients had a clear cell RCC (ccRCC), 16 (15.4) a papillary RCC and 5 (4.8%) a chromophobe RCC. Twenty-one patients had systemic disease (M1) at the time of diagnosis. Clinical follow-up data, as annually assessed survival time was available for all patients. The median follow-up time of all cases was 30 months, ranging from one to 47 months. 21 of the patients died from renal cancer. The pT status was as follows: pT1 – 53 (51.0%), pT2 – 3 (2.9%), pT3 – 45 (43.3) and pT4 – 3 (2.9%). Ten patients (9.6%) had pathologically confirmed nodal metastases (pN1 = 2, pN2 = 8). 50 (48.1%) patients had no nodal metastases (pN0). For 44 (42.3%) patients no lymph nodes were histologically examined (pNx). Tumour grades were G1 – 11 (10.6%), G2 – 74 (71.2%), G3 – 15 (14.4%) and G4 – 4 (3.8%) respectively.

### Tissue Micro Array construction

A tissue-micro-array (TMA) was constructed to represent 108 cases, as previously described [10,11]. The tissue arrayer was purchased from Beecher Instruments (Woodland, USA). The punch diameter was 0.6 mm with each case being represented by two tumour and two normal kidney cores. Four cases were lost during immunohistochemistry processing. All statistical analyses were performed using the 104 cases with GOLPH2 staining available. Matched normal kidney tissue was available for 97 cases.

### Immunohistochemistry

The TMA blocks were freshly cut (3 μm) and mounted on superfrost slides (Menzel Gläser). Immunohistochemistry was conducted with the Ventana Benchmark automated staining system (Ventana Medical Systems, Tucson, AZ) using Ventana reagents for the entire procedure. To detect GOLPH2, a commercially available antibody (mouse monoclonal, clone 5B10, Abnova Corporation, Taipei, Taiwan, catalog number H00051280-M06, dilution 1:1000 was diluted in a Ventana diluent. For primary antibody detection we used the UltraVIEW™ DAB detection kit using the benchmarks CC1m-heat induced epitope retrieval. Slides were counterstained with hematoxylin, dehydrated and mounted.

### Evaluation of the immunohistochemical stainings

The immunostainings were evaluated by two genitourinary pathologists at a multiheaded microscope. The staining intensity was determined by the two pathologists using a four-tier grading system (0 = negative, 1 = weak, 2 = moderate and 3 = strong staining intensity). To achieve a greater uniformity of the evaluation, the first step was to construct a panel with four illustrative examples pictures, of which a hardcopy lay next to the microscope. We used a 10% threshold to determine positivity, irrespective of the intensity grade. Only tumours without any GOLPH2 immunoreactivity or with staining of less than 10% of the tumour cells were considered negative. To delineate between low and high levels of GOLPH2 expression, tumours with moderate to strong GOLPH2 expression (2&3) and tumours with none to weak (0&1) staining intensity were lumped.

### Statistical analysis

Statistical analysis was performed using SPSS, version 15.0. Fisher's exact test, χ^2^-tests were applied to assess the statistical significance of the associations between GOLPH2 expression and clinico-pathological parameters. Univariate survival analysis was carried out according to Kaplan-Meier, differences in survival curves were assessed with the Log rank test. P values < 0.05 were considered significant.

## Results

**Figure 1:**
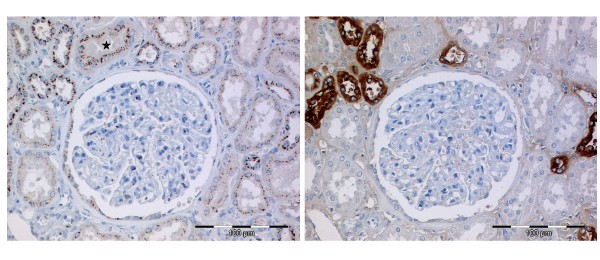
**GOLPH2 immunohistochemistry in normal renal tissue**. **A/B **GOLPH2 expression in normal renal tissue with the typical perinuclear granular staining pattern being more prominent in the distal tubules, although occasionally proximal tubules (star) were also strongly positive (**A**). Staining with Tamm-Horsfall protein in a subsequent tissue section validates the localization of GOLPH2 (**B**).

GOLPH2 immunostaining was easy to evaluate with the typical cytoplasmic and in most cases perinuclear accentuated staining pattern. In normal renal tissue we detected GOLPH2 with its characteristic fine granular staining pattern more often and with a stronger staining intensity in the distal tubules than in proximal tubules (Figure 1). For 97 cases matching normal renal tissue was evaluated. None of these cases were completely negative for GOLPH2, 8 cases were scored 1+ and 89 cases 2+. Renal glomeruli were often completely negative or displayed only few cells with weak to moderate positivity.

**Figure 2:**
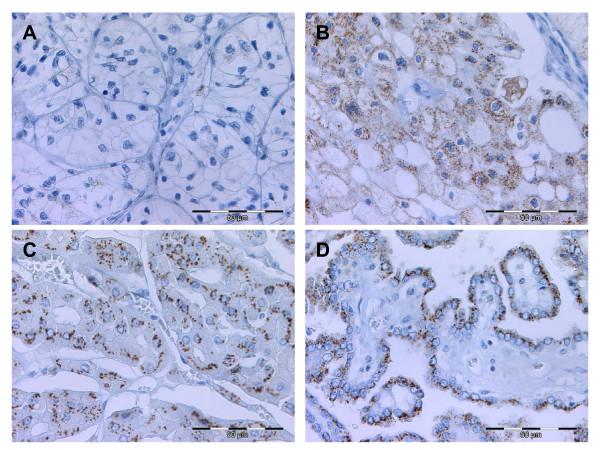
**GOLPH2 immunohistochemistry in renal cell carcinomas**. GOLPH2 expression in clear cell RCC with a negative (**A**) and a strongly positive (**B**) case. All chromophobe (**C**) and the vast majority of the papillary RCC (**D**) were displayed strong immunohistochemial GOLPH2 stainings.

Staining intensities of GOLPH2 in RCC were: negative – 29 (27.9%), 1+ – 30 (28.8%), 2+ – 34 (31.5%) and 3+ – 11 (10.6%). GOLPH2 positivity was significantly higher in papillary and chromophobe RCC if compared to clear cell RCC (Table 1, Figure 2). Other associations or correlations (data not shown) with clinico-pathological parameters could not be detected.

**Table 1:** Associations (χ^2^-tests) between the protein expression GOLPH2 in renal cell cancer and clinico-pathological parameters (percentages in brackets)

	**Total**	GOLPH2 low*	GOLPH2 high*	**p-value**
**All cases**	104 (100)	59 (56.7)*	45 (43.3)*	

**Gender**				0.475
men	81 (77.9)	44 (54.3)	37 (45.7)	
women	23 (22.1)	15 (65.2)	8 (34.8)	

**Age**				0.236
≤ 62	56 (53.8)	35 (62.5)	21 (37.5)	
>62	48 (46.2)	24 (50.0)	24 (50.0)	

**Histology**				<0.001
clear cell	83 (79.8)	58 (69.9)	25 (29.1)	
chromophobe	5 (4.8)	0 (0.0)	5 (100.0)	
papillary	16 (15.4)	1 (6.3)	15 (93.7)	

**pT-status**				1.000
pT1	53 (51.0)	30 (56.6)	23 (43.4)	
pT2/3/4	51 (49.0)	29 (56.9)	22 (43.1)	

**pN-status**				0.455
pN0	50 (48.1)	30 (60.0)	20 (40.0)	
pN1	10 (9.6)	6 (60.0)	4 (40.0)	
pNx	44 (42.3)	23 (52.3)	21 (47.7)	

**Fuhrman grade**				0.347
G 1	11 (10.6)	8 (72.7)	3 (27.3)	
G 2	74 (71.2)	41 (55.4)	33 (44.6)	
G 3/4	19 (18.2)	10 (52.6)	9 (47.4)	

**Metastasis**				0.389
M0/x	83 (79.8)	46 (55.4)	37 (44.6)	
M1	21 (20.2)	13 (61.9)	8 (38.1)	

**Figure 3:**
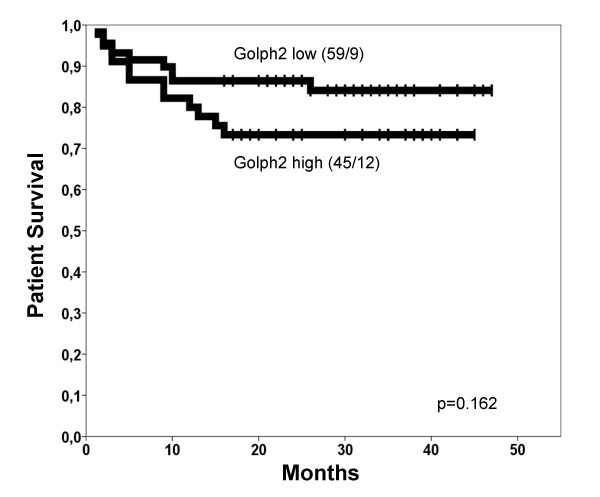
**Kaplan-Meier survival curve for GOLPH2**. Tumours with high GOLPH2 expression (bold line) revealed a slight but insignificant trend for shortened patient survival times if compared to those with low GOLPH2 expression (dotted line). The number of cases/events (deaths) is given in brackets.

The univariate survival analyses demonstrated highly significant p-values for the established tumour markers pT-status, Fuhrman grade, nodal status and distant metastasis (Table 2). Gender and age were no prognosticators for patient survival (data not shown). For GOLPH2 there was no statistically significant prognostic value for patient survival detectable (Table 2), although in the Kaplan-Meier curve a weak trend for longer survival times of patients with GOLPH2 negative tumours was apparent (Figure 3). Importantly, it nearly reached significance (p = 0.057) in the subgroup of clear cell RCCs.

**Table 2:** Univariate survival analysis (Kaplan-Meier)

**Characteristic**	**No. of cases**	**No. of events**	**Two-year survival rate (± SE) in %**	**p-value**
**GOLPH2 expression**				0.162

low	59	9	86.4 ± 4.5	
high	45	12	73.3 ± 6.6	

**pT-status**				< 0.001

pT1	53	2	96.3 ± 2.6	
pT2/3/4	51	19	64.7 ± 6.7	

**Fuhrman grade**				0.001

G 1	11	0	-	
G 2	73	12	84.9 ± 4.2	
G 3/4	19	9	52.6 ± 11.5	

**pN-status**				0.001

pN0	50	10	80.0 ± 5.7	
pN1+	10	7	40.0 ± 15.5	

**Metastasis**				< 0.001

M0	83	8	90.4 ± 3.2	
M1	21	13	42.9 ± 10.8	

Survival times of patients with renal cell cancer according to clinico-pathological characteristics and GOLPH2 protein expression.

## Discussion

This study describes for the first time the expression pattern of GOLPH2 in normal renal tissue and renal cell cancer. GOLPH2 has been described in a variety of tissues, but the most promising results until now have been demonstrated in liver and prostate tissue [2,4,12]. In prostate cancer GOLPH2 is up-regulated on mRNA and protein level in comparison to the normal glandular tissue [8,9,12,13]. In renal cell cancer, this seems not to be the case and the high GOLPH2 expression in normal tissue argues against a use oft this marker for the diagnosis of RCC in histological samples. Whether GOLPH2 levels in serum of RCC patients differ from those of healthy patients has not been tested yet. Further studies of GOLPH2 expression in RCC might focus on its meaning for the different histological subtypes, since our results suggest a different regulation in three most common types of RCC. However, in the present study we did not include any benign renal tumours or other rare histologic types of RCC like sarcomatoid or rhabdoid RCC. We could not detect any associations with conventional tumour and clinical parameters. Since this study cohort was of medium size with most of the cases being clear cell RCC this study cohort was too small to detect a possible prognostic value or subtle clinico-pathological associations for chromophobe and papillary RCC. Still, the different expression patterns of GOLPH2 in the three RCC subtypes were surprising. Especially the difference between papillary and clear cell carcinomas is notable since generally both were thought to be derived from the proximal tubules of the kidney. Given the strong expression of GOLPH2 in normal renal epithelia, two possible explanations for the GOLPH2 down-regulation are possible. GOLPH2 expression might either be lost early in clear cell carcinogenesis and re-expression of GOLPH2 could represent a molecular correlate of tumour de-differentiation. This could explain the pronounced trend in the Kaplan Meier curve of clear cell RCC, which nearly reached statistical significance. On the other hand there was no positive association with the Fuhrman grade. Very recently, Wright et al. provided evidence for a relevant role of GOLPH2 for the integrity of renal and hepatic tissues in a mouse model with expression of a truncated GOLPH2 form [14].

In hepatocellular carcinoma (HCC), GOLPH2 had higher serum levels if compared to healthy individuals and has been proposed as a novel serum marker of HCC [6,15]. However, since GOLPH2 expression in a liver cell line could also be up-regulated by viral (adenovirus) infection, it seems reasonable to assume that either an inflammatory a neoplastic process might be able to trigger GOLPH2 expression in this tissue type [5,16].

Even though in a previous study from our study group (manuscript in preparation) we were able to confirm the GOLPH2 expression in HCC, we also detected GOLPH2 in various other malignancies on a multi-tissue-micro-array which argues against a liver-specific relevance of this biomarker.

In summary, this first systematic analysis of GOLPH2 in renal cell cancer describes that GOLPH2 expression is not restricted to liver or prostate cancer but is also found in a higher proportion of RCC, although no direct diagnostic or prognostic value of GOLPH2 as a tissue biomarker could be confirmed.

## Conclusion

In this tissue-micro-array-based immunohistochemistry study a differential expression of GOLPH2 in normal and malignant renal tissue was demonstrated. While distal tubular epithelia were mainly strongly positive for this marker, other parts of the nephron and the majority of the clear cell RCC were negative. The diagnostic use of the rather constant positivity of papillary and chromophobe RCC is limited since one third of the clear cell carcinomas were also positive for GOLPH2. No other associations with conventional clinico-pathological characteristics were found and no prognostic value of GOLPH2 was demonstrated which limits the diagnostic value of GOLPH2 in RCC.

## Competing interests

The authors declare that they have no competing interests.

## Authors' contributions

FRF coordinated the study, performed immunohistological and statistical analyses and wrote the paper. MOR contributed to statistical analyses and revised the paper. MD and KJ provided samples and clinico-pathological data and supported statistical analyses. HM provided logistical support for the study and revised the paper. GK conceived and coordinated the study, performed immunohistological and statistical analyses and revised the paper.

## Pre-publication history

The pre-publication history for this paper can be accessed here:


